# Reducing Avalanche Build-Up Time by Integrating a Single-Photon Avalanche Diode with a BiCMOS Gating Circuit

**DOI:** 10.3390/s24237598

**Published:** 2024-11-28

**Authors:** Bernhard Goll, Mehran Saadi Nejad, Kerstin Schneider-Hornstein, Horst Zimmermann

**Affiliations:** Institute of Electrodynamics, Microwave and Circuit Engineering, TU Wien, Gusshausstrasse 25/E354-02, A-1040 Wien, Austria; mehran.nejad@tuwien.ac.at (M.S.N.); kerstin.schneider-hornstein@tuwien.ac.at (K.S.-H.); horst.zimmermann@tuwien.ac.at (H.Z.)

**Keywords:** single-photon avalanche diode, SPAD, avalanche transients, gating circuit, CMOS, BiCMOS

## Abstract

It is shown that the integration of a single-photon avalanche diode (SPAD) together with a BiCMOS gating circuit on one chip reduces the parasitic capacitance a lot and therefore reduces the avalanche build-up time. The capacitance of two bondpads, which are necessary for the connection of an SPAD chip and a gating chip, are eliminated by the integration. The gating voltage transients of the SPAD are measured using an integrated mini-pad and a picoprobe. Furthermore, the gating voltage transients of a CMOS gating circuit and of the BiCMOS gating circuit are compared for the same integrated SPAD. The extension of the 0.35 μm CMOS process by an NPN transistor process module enabled the BiCMOS gating circuit. The avalanche build-up time of the SPAD is reduced to 1.6 ns due to the integration compared to about 3 ns for a wire-bonded off-chip SPAD using the same $n^{+}$ and p-well as well as the same 0.35 μm technology.

## 1. Introduction

The avalanche build-up time of avalanche photodiodes (APDs) operated in the linear mode is known to reduce the bandwidth and to increase the rise/fall times of the photocurrent compared to PIN photodiodes [1,2,3]. As a consequence, the maximum data rate of APD receivers is lower than that of PIN photodiode receivers for equal absorption layer thickness [4]. To improve the sensitivity of optical receivers above that of APD receivers, SPAD receivers came into the play. SPADs [5] are operated in the Geiger mode, i.e., above the breakdown voltage of the device, to enable detection of single photons. They, therefore, need an excess bias voltage $V_{EX}$ and the higher $V_{EX}$ is, the higher the photon detection probability is. Since the first introduction of a SPAD receiver with a sensitivity of −31.7 dBm at 100 Mb/s [6], their performance was increased to −49.9 dBm at 400 Mb/s [7], both with thin $p^{+}$/n-well SPADs and 450 nm light. Since 4-PAM was used to extend the data rate to 500 Mb/s (with a sensitivity of −46.1 dBm [7]), a limitation of the data rate in on–off keying of about 400 Mb/s seems to exist. There are other limitations like clock distribution and delay on metal lines, but we took these results as motivation to investigate the avalanche build-up of our thick $n^{+}$/p-well/$P^{-}$/$P^{+}$ SPAD using a thick $P^{-}$ absorption zone, which makes our SPAD better appropriate for longer wavelengths of red and near-infrared light.

Before diving into the details, the state of the art of avalanche build-up of SPADs shall be introduced. Monte Carlo simulations were used to investigate the development of the avalanche current with time. In [8], the maximum avalanche currents of about 8 mA were reached after about 0.5 ns to 0.7 ns (dependent on the location of photon absorption within the device). In other Monte Carlo studies with thin SPADs, the maximum avalanche currents were achieved in less than 0.1 ns [9,10,11]. In [12], a strong dependence of the avalanche build-up time on the thickness of the multiplication region (0.1 μm to 1 μm) from 0.2 ns to 3 ns was reported.

Measurements of the avalanche build-up time were performed in only a few publications to the best of our knowledge. The rise time of the avalanche current was found to be between 0.7 ns and 1.25 ns for decreasing the number of photons from 10,000 to 1 [13]. The transients of the anode voltage of a small integrated SPAD were measured using an integrated buffer (which was unfortunately not described) on the same chip and avalanche build-up times from about 1 ns to 2 ns were reported [14]. The focus of many studies was on the investigation of SPAD jitter. However, one of these studies also reported the direct measurement of the avalanche current with regard to dependence on time at a 50-Ω resistor integrated together with a passively quenched SPAD [15]. According to the reported voltage drop across this resistor, the maximum avalanche current was larger than 4 mA and its rise time was above 0.5 ns. We investigated the avalanche build-up time using a picoprobe to measure the cathode voltage during self-discharging of an off-chip SPAD on one of the two bondpads, one of which was present on the SPAD chip and one was on the gating chip needed for charging the SPAD again [16]. The avalanche build-up time of a wire-bonded SPAD using the same $n^{+}$ and -well as those of the SPAD investigated here was 3 ns determined with a CMOS gating circuit.

Here, we integrate the SPAD together with a BiCMOS gating circuit to reduce the capacitance and to reduce the avalanche build-up time by about a factor of two. We add a mini-pad to be able to measure the voltage transient during self-discharging of the SPAD. From the voltage transient, we derive the time dependence of the avalanche current. Furthermore, we will show that the integration of the SPAD is more important than using BiCMOS instead of CMOS, if focusing on the avalanche build-up time. In addition, it is suggested that the BiCMOS technology will have some advantages for SPAD receiver applications.

## 2. Materials and Methods

### 2.1. Gated Receiver

SPADs enable optical receivers with improved sensitivity [7,17,18]. There are two possibilities for operating the SPAD [19]. The first one is to use active quenching and resetting circuits [20,21]. There, the SPAD is charged to the excess bias and left floating until a photon is absorbed and the avalanche starts. Then, the active quenching circuit switches the SPAD back to the breakdown voltage. After a certain dead time, the SPAD is charged to the excess bias again (this is called resetting). The second possibility is to gate the SPAD [19,22], i.e., to switch it periodically to the excess bias and quench it independent of photon absorption back to the breakdown voltage. This can be achieved with a digital clock, whereby the active phase (with excess bias) can be made shorter than the off-phase (SPAD is biased only with the breakdown voltage). The dark count rate can be much smaller than with an active quencher, especially if this duty ratio is made small. Here, we decided to realize a gated SPAD receiver, motivated by the small gap to the quantum limit reported in [23]. Furthermore, for characterizing the SPAD with respect to avalanche build-up time, an active quenching circuit is not appropriate, because it would not allow us to measure the self-discharging of the SPAD, where it is necessary that the SPAD is not being discharged by a transistor/circuit.

**Figure 1:**
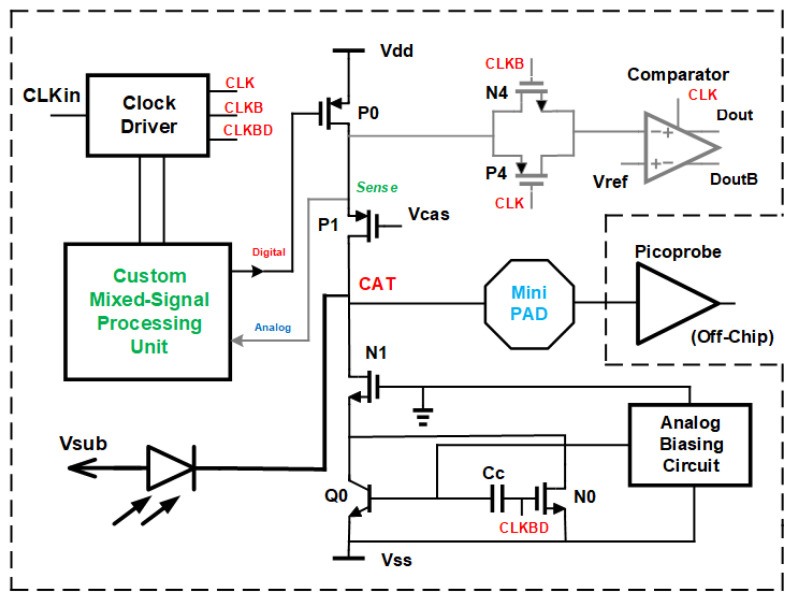
Circuit diagram of BiCMOS receiver with integrated SPAD.

We modified the gated, cascoded CMOS SPAD receiver presented in [16] by exchanging the quenching N-type MOSFET in the cascoded switch with a bipolar NPN transistor. This was motivated by the higher transconductance of bipolar transistors compared to that of MOSFETs [24,25]. The simplified circuit diagram of the resulting BiCMOS SPAD receiver is presented in Figure 1. Transistor Q0 is the bipolar NPN quenching switch. The P-channel MOSFET P0 is the charging switch. Q0 and P0 are cascoded with N1 and P1, respectively, to increase the voltage swing at the cathode of the SPAD to twice the nominal supply voltage of 3.3 V of the 0.35 μm BiCMOS process used, i.e., to 6.6 V. This 6.6 V can be used as maximum excess bias voltage of the SPAD in order to achieve a high photon detection probability. An “analog biasing circuit” (see Figure 1) prebiases the NPN transistor to obtain highest switching frequencies of up to 1000 MHz [26]. Here, we do not use the complete receiver. Only the clock and the cascoded gating switch are necessary to charge the SPAD to breakdown voltage plus excess bias (with P0 and P1), let the SPAD float during the positive gate phase (this is the gate window, in which the SPAD can detect a photon), and to switch the SPAD off with Q0 (plus N0) and N1 until the next charging will be performed. The other components of the front end of the receiver add parasitic capacitance to the cathode node CAT. This parasitic capacitance determines the avalanche build-up time. For the characterization of the avalanche build-up time of the SPAD, we integrated a mini-pad of octagonal shape (in metal layers M3 and M4) with a diameter of 46 μm and an opening of 36 μm inside the passivation layer.

**Figure 2:**
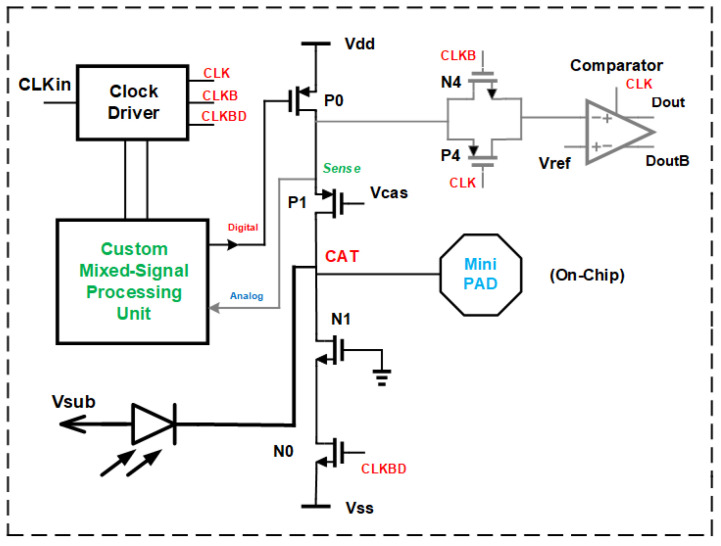
Circuit diagram of CMOS gating circuit.

Since we use two changes (integration of SPAD and using BiCMOS instead of CMOS technology) when compared to [16], we should investigate how the CMOS gater of [16] will perform with the integrated SPAD. To compare a bipolar transistor for switching off the SPAD with a CMOS switch, the CMOS gating circuit in [16] was post-layout simulated with the same on-chip SPAD model, but including a mini-pad for probing and removing a bondpad to have same conditions. Figure 2 shows the appropriate gating circuit in the 0.35 μm CMOS.

**Figure 3:**
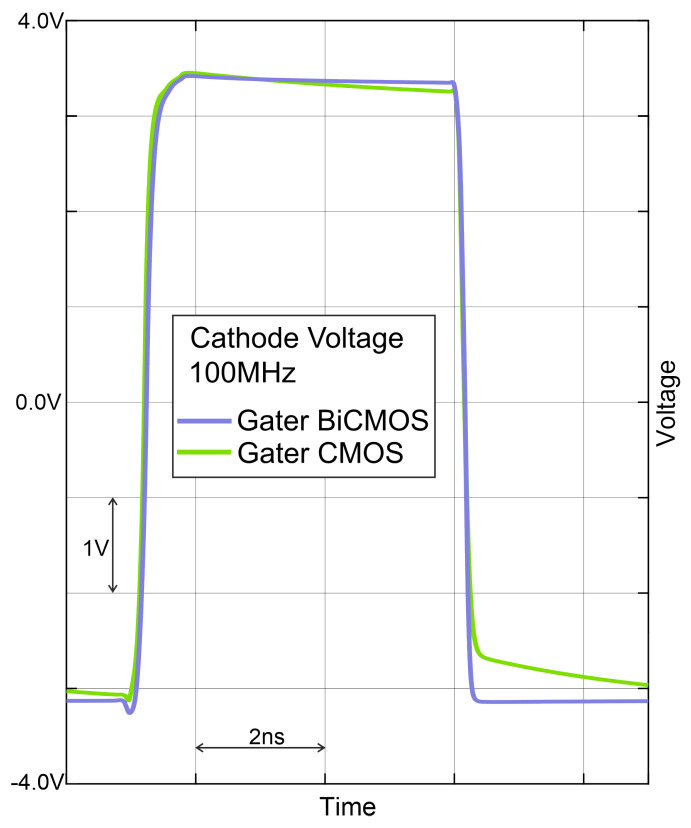
Post-layout simulated transients of 6.6 V gating pulses.

Instead of the bipolar transistor with dynamic coupling at the base and circuitry for analog (pre)biasing, an N-channel MOS transistor N0 is placed, which is switched on and off by the gater clock. The results of the comparison with a post-layout simulation of switching the cathode voltage of the SPAD are depicted in Figure 3. For gating with the bipolar transistor, rise and fall times (10–90%) of 251 ps and 162 ps, respectively, were determined. Instead, the CMOS gater had rise and fall times of 236 ps and 185 ps, respectively. It can be seen that the fall time (quenching time) is more than 10% shorter with the bipolar quenching switch and that the sum of rise and fall times is slightly better for the bipolar gating circuitry. Another advantage is the better capability of the bipolar transistor of pulling down the cathode potential to $V_{ss}$. There exists a slow settling to $V_{ss}$ for the CMOS gater. It originates from parasitic coupling via parasitic capacitances from and to the gate of cascode transistor P1. However, P1 is implemented in both schematics. But due to the “stronger” NPN transistor, these parasitic capacitances do not matter.

**Figure 4:**
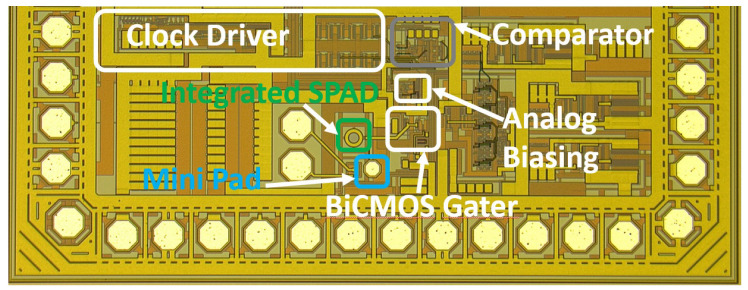
Chip photo of BiCMOS SPAD receiver.

Because of the above mentioned advantages and because a bipolar comparator enables a much higher data rate of the SPAD receiver, only the chip with the bipolar transistor for switching was fabricated in the 0.35 μm XO035 CMOS technology of XFAB inclusive in the bipolar NPN transistor process module (making the CMOS process to a BiCMOS process). The chip photo is shown in Figure 4. The chip has a size of 1.88 × 0.7 ${\mathrm{mm}}^{2}$ with an active area of about 0.045 ${\mathrm{mm}}^{2}$ (SPAD, mini-pad, gating switch, analog (pre)biasing, comparator). Without mini-pad and an optimized layout, however, the SPAD receiver should fit into an area of 200 × 200 μ$m^{2}$.

### 2.2. SPAD

**Figure 5:**
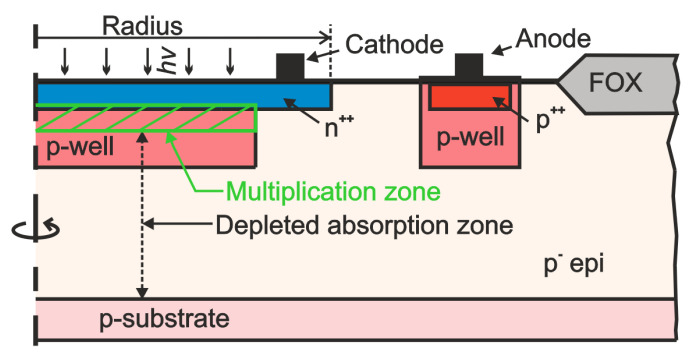
Schematic cross section of integrated SPAD.

The integrated SPAD uses a highly doped ($n^{+}$) cathode and the p-well of the 0.35 μm technology as well as a highly doped p-bulk wafer with an about 12 μm thick low-doped epitaxial layer (see Figure 5). The electric field extends from the $n^{+}$/p-well junction to the p-substrate already at above 19 V. This makes this SPAD a so-called reach-through SPAD [27]. The thick fully depleted $p^{-}$ epitaxial layer acts as absorption zone distinguishing this SPAD by a high photon detection probability for red and near-infrared light. The multiplication zone is located inside the p-well. The p-well and the $n^{+}$ cathode are the same as in the SPAD introduced in [16,23]. But instead of the n-well guard ring in [16,23], a virtual guard ring is used here (see Figure 5). The $n^{+}$ cathode has a radius of 15.3 μm and that of the p-well is 14.5 μm, i.e., the width of the virtual guard ring is 0.8 μm. According to device simulations with ATLAS [28], the capacitance of the SPAD is 15 fF at the breakdown voltage. The breakdown voltage of the SPAD was about 27 V [29]. The motivation for the virtual guard ring is a (slightly) smaller capacitance and a larger effective light sensitive area [30].

The avalanche build-up of the SPAD with the same p-well and $n^{+}$ cathode, but with an n-well guard ring was characterized in [16] with diameters from 50 μm to 400 μm. The SPADs were located in separate chips, which were wire-bonded to a CMOS gating chip using the same 0.35 μm technology. Therefore, two bondpads added their capacitances to the CAT node. The capacitances of the CAT nodes for these SPADs ranged from 0.84 pF to 2.2 pF [16]. To be able to gate SPADs up to 400 μm, the gating switch contained rather wide MOS transistors, which lead to the rather large CAT node capacitances. The fall time of the avalanche transient of the 50 μm diameter SPAD was about 10 ns. The maximum of the avalanche current was achieved after about 3 ns.

## 3. Measurement Results

The BiCMOS gater chip with the integrated SPAD was wire-bonded to a PCB, which was mounted on a Peltier cooler. This setup was built into a wafer prober to be able to measure the cathode voltage with a model 35 picoprobe on the mini-pad. Performing the measurements inside a wafer prober corresponds essentially to measurements in the dark, although a few photons might have been present due to minor leaks of light. With the help of the Peltier cooler, the temperature of the PCB with the chip was regulated to 25 °C. The output of the RF amplifier of the picoprobe with a bandwidth of 26 GHz was connected to a fast real-time oscilloscope (Keysight MSOV204A Mixed Signal Oscilloscope with 20 GHz bandwidth and at maximum an 80 GSa/s sampling rate), with which a lot of periods of the gater clock frequency could be stored for later processing.

**Figure 6:**
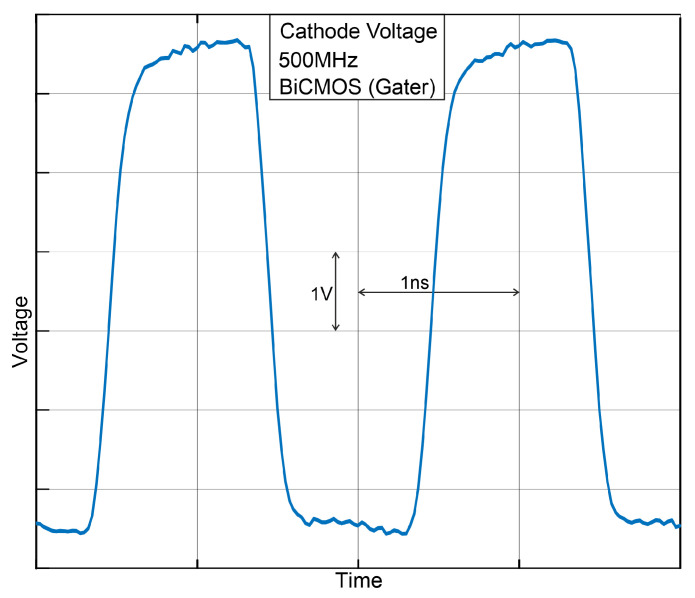
Measured cathode voltage transient at 500 MHz.

First, we clocked the (BiCMOS) gating circuit with 500 MHz, although the BiCMOS gater is capable of switching the cathode of the SPAD with a maximum clock frequency of 1 GHz. With 1 GHz, however, the active gate interval would be only 0.5 ns, which would be too short for a sufficient avalanche build-up according to our experience. The waveform measured at the mini-pad in the wafer prober is shown in Figure 6. No photon absorptions or dark counts are visible. The measured rise and fall times (10% to 90%) of the switched cathode voltages amount to 237 ps and 188 ps, respectively, compared to the post-layout simulated values of 251 ps and 162 ps. These values verify the very high switching speed that was aimed at.

**Figure 7:**
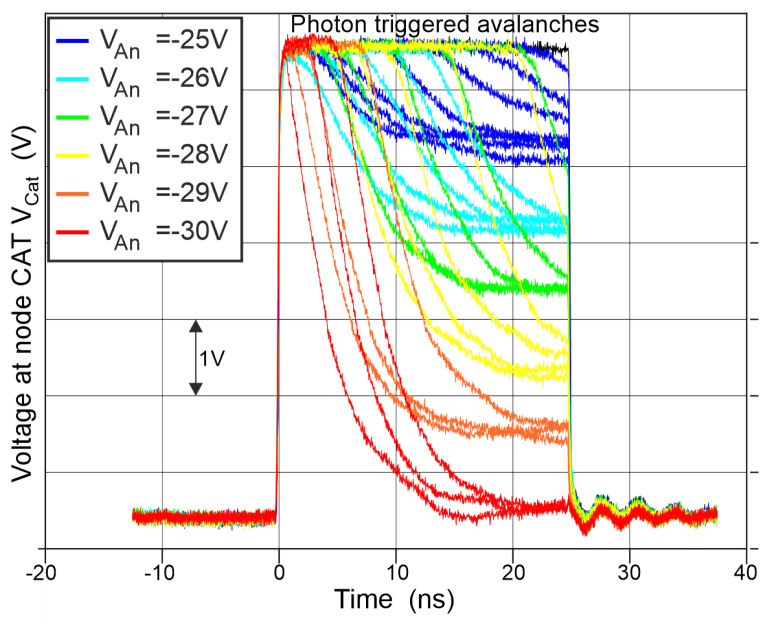
Measured and overlaid self-discharging transients from dark counts.

**Figure 8:**
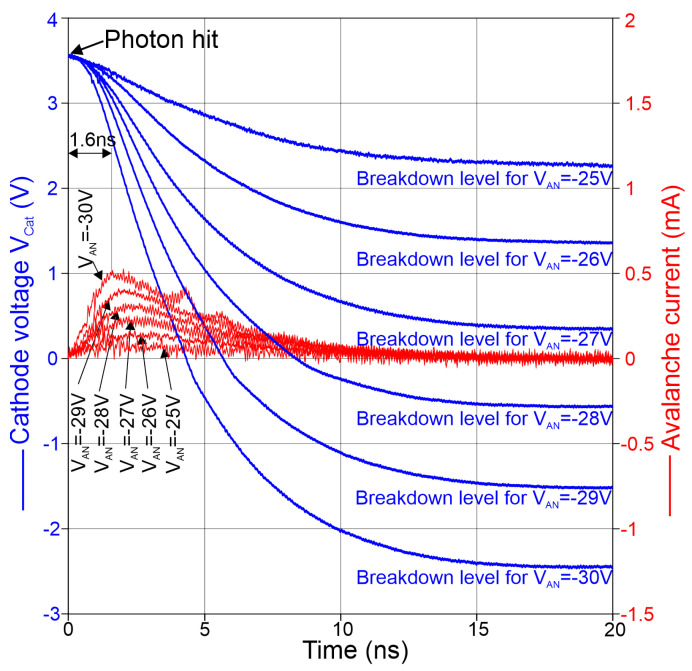
Transients of measured voltage at the CAT node (**blue**) and derived avalanche current (**red**) by dependence on time.

Figure 7 shows the measured cathode voltage of the SPAD, where several clock cycles with 50 ns periods each have been overlayed. The real function of the gating circuit is that during gating action only one avalanche can occur within a clock cycle, which counts for a photon hit. In Figure 7, several avalanche events are combined due to overlaying of many clock cycles. It can be observed that avalanches from dark counts or photon hits with subsequent triggering of an avalanche are time distributed over the clock phase, where the SPAD is set for detecting photons. The different positions of occurring avalanches within the gate window are due to randomly incident photons or randomly happening dark counts. Depending on the excess bias, which can be adjusted between 0 V and 6.6 V with the help of the substrate voltage, the SPAD can discharge itself only to the voltage level, which is defined by the breakdown voltage of the SPAD. Observing this cathode voltage close to the end of the gating window allows us to determine its breakdown voltage (please see below). This means that the SPAD quenches itself within the gating window, if the photon is absorbed during the first part of the gating window. Different voltage levels for the anode $V_{An}$ of the SPAD, which corresponds to the substrate voltage, are marked in different colors. A larger excess bias results in a larger voltage drop during avalanche. The voltage level of the cathode at the end of avalanche discharging is defined by the breakdown voltage $V_{BR}$ (see Figure 7 and Figure 8). A distinct excess bias $V_{EX}$ for the SPAD is defined by the substrate voltage $V_{sub}=V_{An}$ with the relation $V_{EX}$ = $V_{dd}$− $V_{sub}$− $V_{BR}$ (note that $V_{sub}\;=\;V_{An}$ is negative, so $V_{SPAD}$ = $V_{CAT}$ + $|V_{sub}|$; note also that $V_{dd}$ = +3.3 V and $V_{ss}$ = −3.3 V). During avalanche discharging action $V_{SPAD}$ = $V_{CAT}$−$V_{sub}$ is fulfilled, where $V_{CAT}$ is the cathode voltage level and $V_{SPAD}$ is the cathode–anode voltage. After avalanche discharging has finished, $V_{SPAD}$ = $V_{BR}$ and thus $V_{BR}$ = $V_{CAT}$− $V_{sub}$. By measuring the cathode voltage level of the SPAD, a breakdown voltage of approximately $V_{BR}$ = 27 V was determined. Table 1 shows the relation between excess bias and anode voltage $V_{sub}$.

During the detection phase, the cathode potential of the SPAD (node CAT) is pulled up to $V_{dd}$ and set floating for photon detection. If an avalanche is triggered by a photon or by a thermally generated charge carrier, node CAT will be discharged via the SPAD. This can be used to determine the avalanche current.

(1) $$I\left(t\right)=C_{CAT}*\frac{dV_{CAT}}{dt},$$

where $C_{CAT}$ is the effective capacitance of the node CAT. It is difficult to determine $C_{CAT}$ experimentally, because a measurement has to be performed at the mini-pad when the gating circuitry is active and when the node CAT has been switched to high impedance (being floating), i.e., in the short positive gate period when N0 and P0 must not be conducting. A measurement technique could add additional capacitance to node CAT or influence voltage conditions. On the other hand, a simple extraction of capacitances out of the schematic and layout does not consider exactly how different coupled capacitances affect node CAT. Typically, the extraction considers all parasitic capacitances fully, although many of them are not ending on ground and therefore are only partially effective. Therefore, a post-layout simulation was performed with an ideal resistor instead of the SPAD at the CAT node to extract the effective capacitance out of the exponential discharging at node CAT. From this, an effective capacitance of 375 fF was determined for node CAT. Therefore, we chose 375 fF from this post-layout simulation with additional 15 fF of the SPAD and 50 fF input capacitance of the picoprobe to calculate the current through the SPAD. This resulted in an effective capacitance of $C_{CAT}$ = 440 fF to calculate the avalanche current out of the measured time dependence of $V_{CAT}$.

In Figure 8, the results of the calculation of the avalanche current through the SPAD during avalanche according to Equation (1) for different anode voltages $V_{AN}$ = $V_{sub}$ are depicted. The time dependence of the avalanche current is drawn in red. The first peak corresponds to the maximum current, which occurs after the avalanche is built up. $V_{CAT}$ falls further due to discharging through the SPAD until the avalanche is quenched when the breakdown level is reached. For anode voltages lower than −28 V, a second (small) peak can be observed in the time dependence of the current. This second peak occurs because the effective capacitance $C_{CAT}$ becomes lower during a voltage drop of $V_{CAT}$, and in the transition region around 0 V both cascode transistors N1 and P1 (see Figure 1) are turned off. Further dropping turns on N1 and $C_{CAT}$ is effectively larger again. However, for calculating the current to observe rise of avalanche including peak current, the effective capacitance $C_{CAT}$ for turned on P1 and turned off N1 is relevant.

It is, however, important to say that the integral below a current curve in Figure 8 over time represents the avalanche charge flowing through the SPAD. The afterpulsing probability is proportional to this avalanche charge. It is, however, possible to reduce the avalanche charge flowing through the SPAD by switching the excess bias off early with a short gate window, which means that only the area over a (small) part of the current curves has to be integrated.

**Figure 9:**
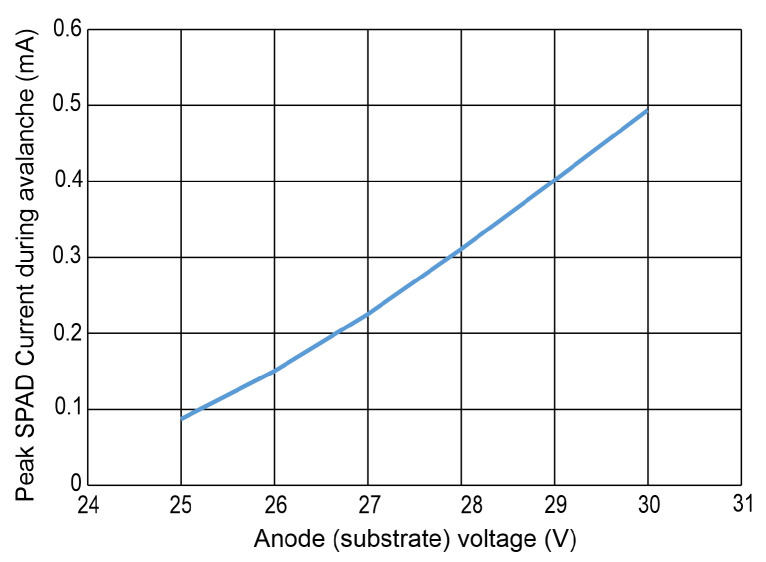
Peak current during avalanche of SPAD vs. substrate voltage.

The peak SPAD current during avalanche in dependence of the substrate voltage is extracted in Figure 9. The peak current is considerably lower compared to [16]. This is because of the much lower input node (CAT node) capacitance in the integrated SPAD receiver investigated here.

**Figure 10:**
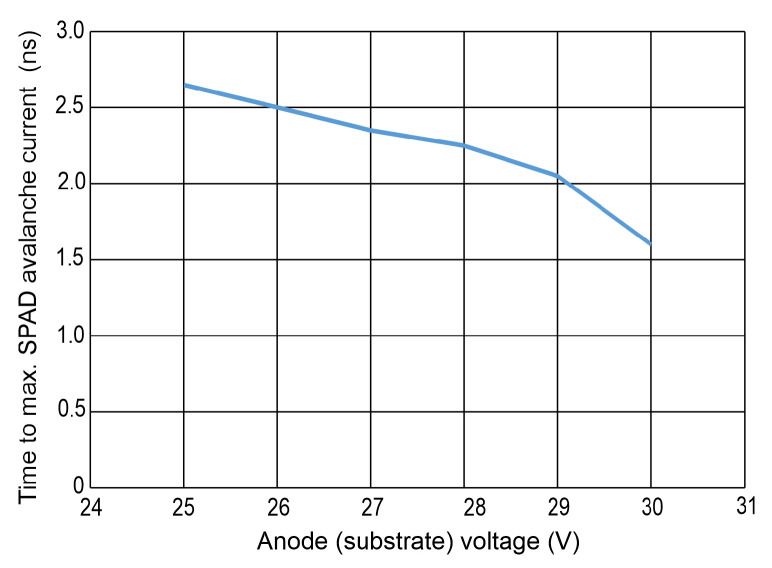
Time of occurrence of the avalanche current maximum of the SPAD vs. substrate voltage.

The time after which the maximum avalanche current occurs is plotted in Figure 10 for the different substrate voltages. For the largest excess bias voltage (largest magnitude of the anode voltage), the peak avalanche current is reached in 1.6 ns. This avalanche build-up time is almost a factor of 2 shorter than in [16], where the SPAD diameter was 50 μm and the SPAD and gater were connected via two bondpads.

**Figure 11:**
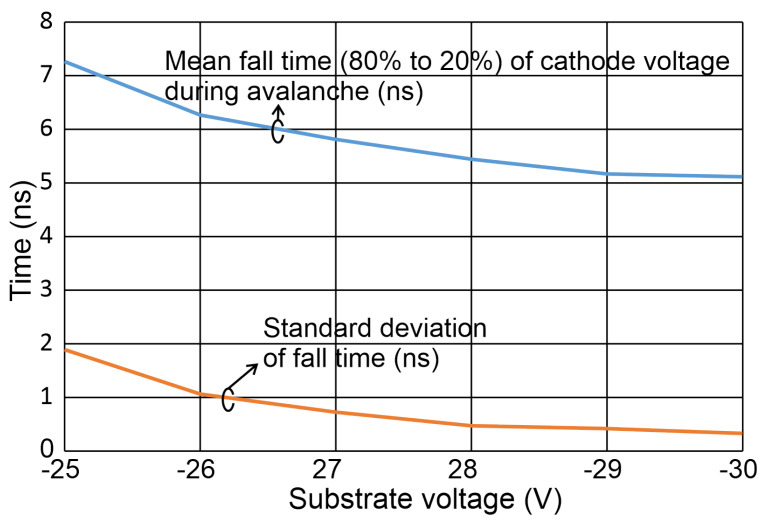
Extracted mean fall times and their standard deviations over substrate voltage.

For a substrate voltage of 30 V, a peak current of 0.49 mA is determined. A measure for the speed of photon detection with the given SPAD–bipolar gating system is the (80% to 20%) fall time of the voltage drop of $V_{CAT}$, which is caused by the avalanche current. When considering an ideal exponential decay of the excess bias, the time constant τ can be calculated by dividing the 80 to 20% fall time by ln(4). A statistical evaluation over more than 2000 samples for each measurement is shown in Figure 11.

For a substrate voltage of $V_{subs}$ = −25 V, the avalanche fall time amounts to 7.3 ns. It drops to 5.1 ns for $V_{subs}$ = −30 V, which corresponds to a larger excess bias of 6.1 V. A fast comparator, however, can detect the avalanche much earlier.

## 4. Discussion

Intuitively, the avalanche should build up faster if the CAT node capacitance is smaller, like a transistor charging or discharging a capacitor. The reduction in the CAT node capacitance is due to the elimination of the two bondpads compared to the experiments published in [16]. Since the bipolar transistor has a better driver capability than MOSFETs, the NPN transistor can be made smaller than an N-channel MOSFET to obtain a smaller capacitance of the collector than the drain–bulk capacitance of the N-channel MOSFET, assuming the same rise/fall times. However, there are the cascode transistors between the quenching switch and the CAT node, which reduce the influence of the capacitance of the quenching transistor. The capacitance of the collector node of the NPN transistor is not visible at the CAT node. The drain-to-bulk capacitance of the cascode transistor N1 is located directly at the CAT node. So, the reduction of the CAT node capacitance in this work is due to the elimination of the two bondpads compared to [16] and not due to the usage of the BiCMOS process extension instead of the CMOS process alone. It also should be noted that the capacitance added by the circuit to the CAT node is much larger than the capacitance of the integrated SPAD alone. If an excess bias voltage of 3.3 V is sufficient, the cascode transistors are not necessary and the bipolar quenching transistor will be more advantageous with respect to a lower input node capacitance. Independent of the excess bias voltage, a bipolar comparator in BiCMOS technology offers a higher transconductance, and in turn more gain and a higher speed than a CMOS comparator [31]. The higher gain allows for a lower detection threshold and the higher speed for a higher data rate of a SPAD receiver.

Another difference to [16] is that the SPAD is somewhat smaller in size compared to the smallest SPAD with 50 μm diameter in that reference. This may reduce lateral growth of the avalanche, shortening the avalanche build-up time. Since the difference in the SPAD radii, however, is not large, this issue will be of less importance.

Due to different locations of photon absorption within the multiplication or absorption zone (the maximum difference of carrier drift time in the thick absorption layer is about 0.3 ns according to device simulations), jitter occurs and due to the statistic process of avalanche multiplication, the avalanche build-up time can somewhat vary. This is expressed by the standard deviation plotted in Figure 11. In addition, the lateral spread of the avalanche included in the so-called instrument response function (IRF) [15] contributes to this standard deviation. However, since the SPAD diameter is rather small, no large jitter from the lateral spread of the avalanche is expected. Therefore, the dominating jitter contribution should be given by the maximum carrier drift time difference in the thick absorption layer.

The lower peak avalanche currents compared to [16] are due to the smaller charge stored on the smaller CAT node capacitance. The smaller CAT node capacitance is therefore important for less afterpulsing and in turn lower bit error ratios of SPAD receivers. In addition, a low detection threshold of the comparator (let us say, e.g., 100 mV below $V_{DD}$) will enable short gate windows that the avalanche cannot build up further in order to keep the avalanche charge flowing through the SPAD smalland in turn reducing the afterpulsing in the SPAD [32] and bit errors in the receiver. If the SPAD is quenched by the quenching switch (Q0), the SPAD is being discharged and the capacitance of the SPAD is being discharged through Q0 and therefore the avalanche charge is kept small.

A “pixel” size of the receiver of 200 × 200 μ$m^{2}$ in 0.35 μm techology will allow for a SPAD receiver array of 16 × 16 with a chip area of about 4 × 4 ${\mathrm{mm}}^{2}$.

## 5. Conclusions

It is shown that the avalanche build-up time of the SPAD integrated in a 0.35 μm (Bi)CMOS technology is almost half that compared to the results reported in [16]. This suggests that this integrated SPAD enables data rates of up to about 500 Mbit/s. The bipolar NPN transistor will be mainly necessary in the comparator to achieve this data rate with an SPAD receiver in 0.35 μm technology. With a small detection threshold of the comparator on the order of 100 mV (which is possible with a bipolar comparator), the avalanche can be detected in less than 1 ns, with the chance of even higher data rates. Due to the dead time being around 10 ns for low enough afterpulsing probability and BER, however, many SPADs will be needed to have still enough SPADs for photon detection, if some fired in previous bits. Table 2 depicts a comparison of fast gating circuits from the literature, which focus mainly on circuit aspects. We restrict this comparison to circuit aspects, since afterpulsing and dark counts mostly depend on the purity of technology, which is used for fabrication of the SPAD. The gating circuit in pure silicon BiCMOS suggested here achieves a much shorter rise/fall time of the gating window than the silicon–germanium BiCMOS circuit [33] and a 6.6 V excess bias instead of 5 V. When we assume a proportional dependence of rise/fall time on excess bias/step height of the gating window, the post-layout simulated values of 480/280 ps reported in [34] for 9.9 V excess bias should be 320/187 ps for 6.6 V excess bias. The rise time of the BiCMOS gating circuit investigated here is about 90 ps shorter. Only with sine-wave gating performed with standard/discrete components was a higher gating frequency (1.4 GHz) reported in [35].

## Figures and Tables

**Table 1:** Corresponding excess bias $V_{EX}$ for a given anode voltage (substrate voltage $V_{sub}$).

$V_{\mathrm{sub}}$ (V)	$V_{\mathrm{EX}}$ (V)
−25	1.1
−26	2.1
−27	3.1
−28	4.1
−29	5.1
−30	6.1

**Table 2:** Comparison of fast gating circuits focusing on circuit aspects.

	Technology	Type of Gating	Max. Frequency	Max. Excess Bias (V)	Rise/Fall Time (ps)
[33]	SiGe BiCMOS, InGaAs/InP SPAD	rectangular	100 MHz (counter)	5 V	300 (20–80%)
[34]	0.18 µm HV CMOS	rectangular	150 MHz	9.9 V	480/280 (10–90%)
[35]	discrete, InGaAs/InP SPADs	differential sine wave for SPAD and dummy SPAD	1.4 GHz	7 V	-
**This Work**	0.35 µm BiCMOS	rectangular	1.0 GHz	6.6 V	237/188 (10–90%)

## Data Availability

All data are published in this article illustrated in figures.
